# The clinical and psychosocial journey of young people engaging with early intervention psychosis services: qualitative study

**DOI:** 10.1192/bjo.2025.10848

**Published:** 2025-10-23

**Authors:** Patrick Caldwell, Nicholas Glozier, Tacita Powell, Katrina Conn, Rochelle Einboden, Niels Buus, Ellie Brown, Isabella Choi, Alyssa Milton

**Affiliations:** School of Rural Health, Faculty of Medicine and Health, University of Sydney, Australia; Sydney School of Medicine (Central Clinical School), Faculty of Medicine and Health, University of Sydney, Australia; The University of Sydney and Australian Research Council (ARC) Centre of Excellence for Children and Families over the Life Course, Australia; Adolescent Court and Community Team Justice Health and Forensic Mental Health Network, Sydney, Australia; New South Wales Department of Education, Sydney, Australia; School of Nursing, University of Ottawa, ON, Canada; Children’s Hospital of Eastern Ontario (CHEO), CHEO Research Institute, Ottawa, ON, Canada; Susan Wakil School of Nursing and Midwifery, Faculty of Medicine and Health, The University of Sydney, Australia; School of Nursing and Midwifery, Western Sydney University, Australia; School of Nursing and Midwifery, Monash University, Australia; Department of Public Health, Aarhus University, Denmark; Orygen, Parkville, Melbourne, Australia; Centre for Youth Mental Health, University of Melbourne, Australia

**Keywords:** Early intervention psychosis, peer support, psychosocial recovery, self-management

## Abstract

**Background:**

Early Intervention Psychosis Services (EIPS) provide multimodal interventions for young people who are at risk of, or have experienced, a first episode of psychosis. Although recent studies have begun to examine this critical period in a young person’s personal recovery in more depth, little is known about how young people experience EIPS in general, and its influences on their clinical and psychosocial recovery in particular.

**Aims:**

This study aimed to explore young people’s experience of EIPS, specifically the factors that have affected their (a) clinical and (b) psychosocial recovery.

**Method:**

This study purposively sampled 27 young people from a range of backgrounds at 6 community-based EIPS in Australia. Audio-recorded, semi-structured interviews were conducted and reflexive thematic analysis was used to analyse this data-set.

**Results:**

Four themes of how EIPS enabled recovery were identified. The first three - a safe space, unconditional support and active involvement – were foundational to a fourth theme of gradual self-management. In earlier-stage self-management, participants relied on practical supports to make connections and find education and employment opportunities. By later-stage self-management, they had developed the tools to do these things for themselves. Participants’ movement between earlier- and later-stage self-management was connected to their overall EIPS engagement and, for some, to their engagement with peer support.

**Conclusions:**

Providing a safe space, unconditional support and active involvement for clients and their families created the foundational conditions for improved clinical and psychosocial recovery. Peer support programmes, increasing engagement when situational changes such as employment occur and the provision of culturally sensitive care appeared valuable to this process.

Early Intervention Psychosis Services (EIPS) provide multimodal interventions for young people (in general, aged 12–25 years, but age limits may vary by location) who are at risk of, or have experienced, a first episode of psychosis. EIPS aim to (a) reduce any period of untreated psychosis; (b) reduce the risk of transition to full-threshold psychosis; (c) restore functional trajectory; and (d) minimise the impact on the family system.^1^ Early intervention services are designed to intervene at a ‘critical period’^2^ when treatment is likely to be of maximum short- and long-term benefit,^3^ and when clients may still have the benefit of supportive social structures that are crucial to their clinical and psychosocial recovery.^4,5^

## What EIPS offer a young person

Australian EIPS follow guidelines first developed in 1992, which form the basis for much best practice internationally.^1,6,7^ EIPS are delivered in Australia via 2 parallel systems: (a) state-funded EIPS teams operating within geographically bounded local health districts (LHDs) with links to psychiatric hospitals (the state of New South Wales, for example, has 19 EIPS teams at 17 sites);^8^ and, at the time of this study, (b) 6 federally funded headspace centres (some with sub-units, for a total of 14 headspace EIPS nationwide).^9^ A young person entering an EIPS through either system is assigned to a care coordinator (also known as a case manager, with a nursing, psychology, social work or other clinical or allied health background), who is their primary service contact and responsible for both direct support and facilitating other assistance through the service. EIPS also offer medication and psychological therapies, one-on-one and group psychoeducation and assistance towards psychosocial or functional recovery, including education, employment and family support.^1,6^ Individual EIPS resource levels affect whether they offer all of these services under one roof or refer clients on to third parties with whom they have a relationship (e.g. to an employment agency to assist a client in writing a resumé).^9^

## What we currently understand about young people’s experiences of EIPS

Hansen et al’s well-conducted 2018 qualitative meta-synthesis highlighted that the rediscovery of a personal sense of agency was an important moment in a participant’s recovery journey; and with that, with this sense of being able to take control of their recovery, came (for some, at least) the belief that change was possible. The authors also described the importance of personal relationships to the recovery journey, in particular their relationships with EIPS staff, peer groups and family and friends.^10^ Loughlin et al, in a 2020 meta-synthesis, similarly highlight the importance of a service user having a high-quality relationship with their care coordinator in relation to their recovery, and the impact of this relationship on a service user’s sense of agency with regard to their mental health issues.^11^ Nevertheless, research in this field is still in the early stages of exploring the constituent ingredients of these themes; and of working out what it is that service staff do (or don’t do) that is particularly important in building quality relationships, or what it is about a given service that fosters a growing sense of personal agency in a client. There is also very little research in general, and no known qualitative research in particular, that explores the experiences of clients from culturally and linguistically diverse backgrounds, despite a much higher prevalence of psychotic disorders in these groups relative to the general population.^12,13^

Recent studies have begun to examine specific components of a young person’s recovery in more depth. Heijden-Hobus et al explored service users’ personal preferences for care in the acute phase and early recovery period, describing the importance of tranquility during the episode of psychosis as being very important in the acute phase, and the need for information (regarding psychosis itself, and the medications they were taking) as often going unmet in both the acute phase and early recovery period.^14^ Chua et al explored the experience of 12 service users who had disengaged from EIPS, finding complex family dynamics present in the decision to disengage for 8 of the 12 participants (‘Actually it’s one of my cousins who asked me to, if I’m OK already then just stop taking the medicine’).^15^

The authors of the present paper have recently explored how service users experience transitions (into or out of an EIPS, or within-service changes)^8^ and hospitalisations,^16^ finding in both contexts that consistent care coordination and, where relevant, clinical handover were critically important to facilitating continued constructive engagement.

## What this study aims to add

The current paper aims to better understand the journey of young people at EIPS across various stages of clinical and psychosocial recovery, from those diagnosed as at-risk, through recently admitted for first-episode psychosis, to largely self-sufficient. In particular, it aims to understand the interplay between the environment for recovery provided by an out-patient EIPS and those factors highlighted by previous literature as being important to the recovery journey, namely those affecting service users’ engagement, sense of agency and readiness and willingness to move beyond the supports of their service as they make their way in the world. It aims to add to our understanding of what works for young people in EIPS – and what does not – and to inform future practice in this field.

## Method

### Design

This study sat within a larger service evaluation of six EIPS commissioned by the Australian Federal Government.^9^ It comprised 1:1 semi-structured interviews, which were analysed thematically.^17,18^ Acknowledging its limitations, the study is reported in accordance with the consolidated criteria for reporting qualitative research (COREQ),^19^ and this checklist is included in Supplementary File 1 as required. This study drew on a critical realist perspective, which assumes that participants’ accounts reflect both lived experience and underlying social and structural mechanisms. This orientation guided our interest not only in what young people reported, but also in uncovering the conditions and mechanisms that enabled or constrained those experiences. The authors assert that all research contributing to this work complies with the ethical standards of the relevant national and institutional committees on human experimentation, and with the Helsinki Declaration of 1975 as revised in 2013. All research involving human subjects was approved by Sydney Local Health District Human Research Ethics Committee (approval nos X17-0398 and 2019/ETH07234).

### Setting

The study sites were six community-based EIPS located in New South Wales (NSW, five sites) and the Northern Territory (one site), Australia. They included services from three of the new federally funded multidisciplinary, first-episode psychosis/ultra-high-risk headspace services (Western Sydney and Darwin) and three state-funded services in Sydney, one of which, in Western Sydney, covered the same geographical area as the federally funded services. The three geographic locations (inner Sydney, Western Sydney and Darwin) differ in their demographics: inner Sydney is densely populated, with a high student population and a high proportion of employed professionals; Western Sydney is the population centre of a large metropolis and home to a high proportion of people born outside of Australia; and Darwin is a regional city with a large proportion of Aboriginal and Torres Strait Islander peoples, as well as recent immigrants from South-East Asia. In Darwin there is no state-based service. In NSW, when state and federal services operate alongside each other, state-based services generally accept clients presenting with more severe illness.

### Participants

The participants were young people accessing a participating EIPS who were eligible for inclusion if they met the following criteria: (a) aged 12–26 years; (b) nominated by their treating clinician; (c) had been with their respective EIPS for a minimum of 2 weeks; and (d) provided written consent. For participants aged 12–15 years, additional parental or guardian consent was required; for participants aged 16–18 years, additional parental or guardian consent was required if advised by the participant’s treating clinician and/or required by state-specific laws. In total, 27 young people aged 15–27 years (by the time of interview) were recruited into the study. Participant demographic data are detailed in Table 1.

**Table 1:** Participant demographics (*n* = 27)

Demographic	*n*	%
Months in programme (median, 15 months; range, 1–55 months)		
0–14	12	44
15+	15	56
EIPS service type		
State-funded	8	30
Federally funded	19	70
EIPS location		
Inner Sydney	7	26
Western Sydney	15	56
Darwin	5	19
Age at time of interview (years)		
15–19	9	33
20–22	9	33
23–27	9	33
Gender		
Male	15	56
Female	12	44
Other	0	0
Domestic situation		
Family	19	70
Partner/husband/wife	2	7
Friend(s)	2	7
State-appointed carer	1	4
Alone	1	4
Not specified	2	7
Aboriginal or Torres Strait Islander		
Yes	3	11
No	24	89
Not specified	0	0
Language other than English		
Yes	9	33
No	18	67
Education, vocational training or employment		
Yes	21	78
No	6	22
Not specified	0	0
Currently in education (including vocational training)		
Yes	17	63
No	10	37
Currently in employment		
Yes	10	37
No	17	63
Clinical diagnosis		
At-risk	5	19
First-episode psychosis	22	81

EIPS, Early Intervention Psychosis Services.

### Recruitment and consent

Participants were purposively sampled for this study via clinician referral. Clinicians were asked to approach potential participants from diverse backgrounds, including young people from culturally and linguistically diverse backgrounds and those who identified as First Nations people (a person with Aboriginal and/or Torres Strait Islander heritage). Recruitment was checked at the second stage to ensure that no gender/cultural combination exceeded 35%. Clinicians were also asked to nominate clients at different stages of the client journey, namely recent entry into the EIPS (checked via recent completion of an assessment for service eligibility); completion of eligibility for continuing care and case management (which occurs around the mid-stage of the client journey); and eligibility for service and support (which tends to occur in the latter stages of engagement). The researchers fed back to the coordinating clinicians at each EIPS if there were any recruitment gaps, and clinicians checked their entire caseload for eligibility to minimise the potential for gatekeeping and bias.

A two-stage consent process was applied, in which clinicians briefly described the study to potential participants meeting eligibility criteria gaining consent to refer to the researcher. The clinician scheduled the interview for those expressing interest in participating. Prior to interview, participants had the opportunity to review the participant information and consent forms, and to discuss any questions, before giving informed consent. Participants aged 12–18 years required parental/guardian co-consent, with 16- to 18-year-olds’ parental/guardian consent being subject to clinician advice and state-specific laws.

### Data collection

Because this study was part of a national evaluation, data saturation was not assessed. While saturation is commonly used in qualitative health research, in this study, pragmatic sampling targets were guided by evaluation scope and practical constraints rather than by formal saturation criteria. Instead, we relied on *a priori* estimates to guide sample size, detailed elsewhere.^8^ Twenty-eight young people agreed to be interviewed. One withdrew consent following clinician nomination but prior to interview, leaving 27 who completed audio-recorded interviews conducted face-to-face at the EIPS, or via telephone, between December 2019 and May 2020. Two participants requested the presence of a support person, who also participated in the interviews. Interviews were conducted by either a psychologist and qualitative researcher (A.M.) or a psychiatry senior registrar (T.P.), were transcribed via the NVivo Transcription Service then anonymised and checked for accuracy by four members of the research team. The mean interview duration was 57 min (standard deviation 18 min). Transcripts were not returned to participants.

### Analysis

Reflexive thematic analysis was considered the most appropriate tool to achieve the study goal of exploring participants’ experiences. The overarching analysis was inductive (a ‘bottom-up’ identification of themes and patterns in the data) using a mix of semantic and latent coding.^20–22^ Once these themes linking participants’ experiences were identified, it became possible to ask the question ‘what must be true for this experience to occur?’ and, consistent with a critical realist perspective, to theorise the underlying mechanisms and service conditions that enabled or constrained personal recovery. The lead author (P.C.) conducted the analysis using a well-established, six-step process of qualitative analysis, ensuring that all of the data relevant to a young person’s personal recovery had been considered and that a coherent thematic map had been developed, which included themes and subthemes and answered the study’s research questions.^18,22^ Descriptions of the themes, subthemes and codes were developed and captured in a coding framework facilitated in NVivo 12 software for Windows (QSR International Pty Ltd, Burlington, MA, USA; see https://lumivero.com). Analysis was discussed weekly with A.M. Results were triangulated with both past data analysis and the wider study team who had previously analysed the data. The interpretation of participants’ narratives was viewed as reflecting both their lived experience and deeper mechanisms that shape recovery journeys, and hence realist ontology guided our interpretation of how personal, relational and systemic conditions enabled or constrained recovery.

## Results

Four EIPS-related themes were identified in participants’ personal recovery journey: (a) a safe space; participants felt they could engage with EIPS without fear of judgement or of burdening the listener; (b) unconditional support; participants felt that EIPS staff wanted to be there for them; (c) active involvement; participants wanted to feel like partners in the management of their condition; and (d) gradual self-management; participants, to varying degrees, felt they had the tools to form connections of their own in life, work and education. Where participants were actively managing their own condition and making their own way in the world (via social connections, employment and/or education), the study team considered them to be later-stage. Where participants were still in the early stages of doing so, and relied more heavily on the supports of their EIPS as they began to engage in these activities, they were considered to be earlier-stage. Acknowledging the constraints of a single interview to capture participants’ experiences, the authors assessed that 7 participants were earlier-stage and 20 were later-stage. Four of 7 earlier-stage participants and 11 of 20 later-stage participants had been service users for greater than the median of 15 months, indicating that time spent with a service did not automatically correlate with clinical or psychosocial recovery.

### A safe space

Participants described EIPS as a safe space where they could go to discuss what was on their mind without fear of judgement. One subtheme that emerged was comfort, where many participants reported ‘feeling at home’ (participant 8, P8) in a welcoming EIPS environment. The second subtheme was accessibility, where participants thought of EIPS as the place to go when affected by mental illness and, for a small number of participants, the anonymity afforded by an EIPS (compared with school or home environments) meant that they felt greater freedom to fully engage with the service: ‘Well, I guess they have just given me a place where I can speak my mind’ (P13). The third subtheme was impartiality, whereby participants felt that, at EIPS, they could gain the perspective of someone unrelated to them and without worrying that they would be burdening them.

Finally, for a number of participants, these subthemes coalesced in creating a space in their lives that was unique and positioned as an alternative to their home life: ‘They’ll listen to me. So, it’s nice. I don’t have that at home. My mum’s always so busy like I barely have time to talk with anyone. And then all my siblings are younger than me. I don’t want to tell them all my problems and stuff’ (P15). ‘Most participants from culturally and linguistically diverse backgrounds were more likely to describe their experiences of EIPS in these terms (Table 2).

**Table 2:** Foundational themes: a safe space, unconditional support and active participation

Theme	Subtheme	Illustrative quote
A safe space	Comfort	‘I feel at home here. I feel comfortable. I have peace of mind when I talk to someone. You hear [a] more positive message’ (P8).
	Accessibility	‘They are always just one text away’ (P10). ’Oh, I just went to see [care coordinator]. And I was like “Probably going to try to kill myself, hey … Can I go to the hospital?” and she was like “Yeah, alright, let’s go over”’ (P1).
	Impartiality	‘In a broad sense, having someone that I can talk to and I know it won’t make them feel bad if I tell them things, you know what I mean?’ (P6).
Unconditional support	Checking in	‘[EIPS team member] has come out and seen me a couple of times, especially when I was at uni, because I get very stressed about just keeping uni when, like, there were classes, so she would come to my university, we would talk there’ (P3).
	On my side	‘I’ve observed, people here they really care. They look after you’ (P1).
	Tailored to my needs	‘Yeah, um, when I relapse I usually come in at least once a week. Now at the moment I probably come in every three weeks’ time maybe, just to catch up and see how I’m going, just have a chat and that. But yeah, when I relapse I usually see them like every, once a week just to constantly check up on how I’m going’ (P9).
Active participation	I feel like a partner in my own care	‘And they also take into account like what time in my life it is say before starting school, starting a new school year. I’ll probably be a stress, so you know they’ll say “we will lower your medication a couple of weeks into the school term”. You know they take into account as well what I had on in my life, and that was really helpful’ (P25).
Involving my family helps me	‘My mum was just worried that the medication was gonna make me feel worse or that I was going to be addicted to it for life or something. But they let my mum know that when it was time for me to stop, they would like slowly get me off it’ (P15).	

P1, participant no. 1, etc.

### Unconditional support

Participants described the way that EIPS staff in general – and their care coordinators in particular – would check in with them as foundational to their sense of being supported in their journey of personal recovery: ‘It was really nice to have someone who would want to call in and want to check in with me, even if I wasn’t like a family member or friend. It was someone who wanted to check in with me because they were really actually concerned’ (P25).

Participants also described a process of trust-building at their EIPS that ‘takes time’ (P20), and around a third of participants contrasted prior clinical experiences in the community, such as using general practitioner or psychology services, unfavourably with their time at EIPS. A subtheme that emerged highlighted how the unconditional positive regard that participants experienced from EIPS clinical staff, which, for some, had been missing previously, was instrumental in helping develop the sense that they were ‘on my side’ (P1): ‘… as soon as I said what I was experiencing, they took it seriously and they’re just like, “Ok, no, this is how she says she feels”, like, very trusting’ (P3).

A final subtheme that emerged here illustrated the importance of flexibility in the intensity of support proportional to changing participant needs, especially in predicting times of relapse or potential triggers for relapse. Participants generally understood the need for escalated support even when they found it annoying at the time: ‘At the time, it’s not too good because obviously I don’t want to be spoken to, but when they can get a hold of me and I can talk about it, it’s obviously life-changing’ (P27). Tailoring the intensity and focus support enabled accounting for recovery being a non-linear process in which participants experienced less straightforward trajectories or even relapse. Sometimes seemingly positive steps, such as starting a new job, preceded a period where a participant would feel a reduced need for contact with their EIPS, but in retrospect the new-found demands of employment often meant that the opposite was the case. The experience of one earlier-stage participant illustrates this interesting back-and-forth trajectory. He described working as a factory storeman for 8 months: ‘working … it kept me going, it gave me money in my pocket, plus it gives me an opportunity to meet new people’ (P20), then a difficult period in which he was involved in a fight on the street and lost his job due to a work injury. Then:

‘I spent 2 days in my room thinking “what could I do better next time?” And then also headspace came … So it was very refreshing knowing that I got support by my side, that eased me a bit … Knowing that I got support, [if] I need to talk to someone, I need to see someone, what I’m going through, why I cannot sleep during the day, during nights’ (P20).

### Active participation

Participants wanted to feel like a partner in the management of their condition. Although this theme recurred in a variety of contexts, nearly all participants recalled this most readily in the context of discussions around their medication regime. Participants were sensitive to the impression that their input was not being sought or facilitated: ‘I mean, you know, it’s the first time I’ve seen him and he kind of wants to make big medication changes … And it’s like you barely even f**king know me’ (P1). Instructively, many valued their own engagement in their personal recovery equally with the contributions of their treating clinician: ‘We have a conversation about it. It started off heavier, and then I realised I didn’t need certain medication. Then we talked about it again and then steadily as we talked and they saw my progress, I went down in dosage of everything except my lithium’ (P5).

One surprising caveat, reported by a small number of young people, was that potentially over-prioritising participants’ expertise in their condition could detract from the clinician’s role as an expert: ‘I remember one of the registrars asking me when I wanted to lower my dose for medication, and it seemed very arbitrary like if I said yes they’d lower it today like it’s whenever I want and I just thought he should have a better idea than I do. Yeah, it was kind of like a bit too much involvement, too much autonomy almost’ (P18).

A subtheme that emerged here was, where possible, actively involving families in the young person’s care. This was particularly important where there were family-centred obstacles to optimal care (e.g. stigmatising or potentially conflicting attitudes to medication or to mental illness generally). These obstacles were frequently raised by young people from culturally and linguistically diverse families in particular: ‘So my mum was really hesitant with my medication, she used to come to this doctor’s appointment. And she would always ask the doctor, can we reduce the medication? Is it too high? Can we stop the medication? When do we stop it?’ (P19). For these families, culturally sensitive care could involve small, proactive measures: ‘And so we were in the waiting room and my mum would pick those information sheets [in different languages] up and she would read them … So those really helped’ (P19).

When participants and their families described being able to engage with EIPS staff of the same cultural background, connection was easier: ‘I think my dad said that he’s become more aware of a difference between Chinese and Western culture because the family counsellor is also Chinese, so I think sometimes when he talks, they talk in Chinese, the family counsellor would kind of say, like in our culture it’s like this and then here it’s this kind of thing’ (P18). Often, participants described that they and their families had previously experienced feeling excluded from decisions around care and felt more like active participants in EIPS. This brought with it a sense of relief. For families whose EIPS did not yet have staff with whom they felt sufficient connection, family peer support often filled the gap: ‘So they said that my mum should go to a family therapy that will kind of session where the peer supporters run it and they talk to the family about anxiety and psychosis … And so they come to the house and they talk to my mum and they talk to and, you know, they share a common ground where their child is sick. And my mum feels like she’s not alone’ (P19).

### Gradual self-management

As participants reflected on their EIPS’ efforts to help them develop the tools to manage their physical and mental health, and to form connections of their own in life, education and employment, two subthemes emerged: earlier- and later-stage self-management. In the earlier stages, participants reported the cumulative effect of having a safe space, unconditional support and increasingly active participation in their care: ‘They help like, they’ve grown my socialising because they talk to me. Therefore, my self-esteem, confidence. They also improve my interaction with people’ (P11). They also relied on practical measures to reintroduce them to social interactions, including one-on-one communication skill-building and formal and informal peer support; to education, including liaising with educational institutions and helping participants obtain modifications to their study programme; and to employment, including careers advice, job-search assistance and resume-building. This assistance was often mentioned as something that helped participants strengthen their connection to others:

‘Yeah, definitely. I would say, especially with my family as well, they have helped me build more deeper trusting relationships. I have the confidence now to come up to my dad, or my parents, or my brothers to say what I’m feeling. I come up to friends now and teachers as well. I never used to be able to do that. I couldn’t approach anyone about it. And they’ve helped me to approach people about it and helped me, you know, and just taught me how to express my emotions and when I’m in distress to other people and not to be ashamed or scared of that at all’ (P25).

All participants in a later stage of their recovery journey with EIPS described important connections in their life – for some, like P25 above, pre-existing relationships with friends and family were important. For others, new relationships forged via peer connection at their service played an important role in their ongoing recovery (both as relationships in and of themselves and in building the skills of making connections beyond their service). An important aspect of these connections was the relatability of their peers:

‘They have Chill Space, which is like peer support run by other people who have gone through the same thing as me. And so that’s helped me with my social anxiety. I feel much more confident in speaking with other people and like interacting with others that have gone through similar things. But we don’t actually talk about it [psychosis] as often. We talk about it as a friendship, kind of like our friends. We’ve gone through the same thing. We know that. We talk about Christmas time and stuff like that’ (P19).

Importantly, while peer- and other group-based support was often highlighted as an important component of recovery (see Table 3), when participants had a feeling that a given group lacked relatability they were less likely to participate – as noted by one participant who commented on a non-peer-led group advertising:

**Table 3:** Exploring gradual self-management

Theme	Topic	Illustrative quote
Self-management – earlier stage	Mental health	‘But I was able to get special consideration from university, drop a couple of subjects. And spend some time with family, friends, and that just got me back on my feet so I didn’t actually need to go into hospital … I spent a lot of time talking to [clinical psychologist] and [psychiatrist] about it. [Psychiatrist] helped me to understand that, for me at that time, wanting to go to hospital was actually about wanting rest and wanting care, and, and I wasn’t at a stage where going to hospital would be able to provide’ (P4).
	Socialisation	‘It’s really helped with me in being more confident in interacting with other people since Chill Space, which was pretty new, started only this year. So since then, like the past couple or three or four years, I haven’t been as social and I wasn’t very, like, I was very anxious socially. I didn’t have that many friends. But I think since Chill Space, which was a peer-supported-run group that really helped me open up. So I felt like it helped me make friends easily’ (P19).
	Education	‘[EIPS] was great in helping me in a sort of recovery, you know, talking to the school about going back to school’ (P25).
	Employment	’So the same person that would take me [to Centrelink] would teach me about how to write a resume. They also took me to Centrelink and got me my youth allowance’ (P19).
Self-management – later stage	Mental health	‘… Because from my experience as someone with mental health [issues], I can, you know, in mental health conditions, I never got better until I started to implement things myself. Throughout it was like, yes, the medication helped. Yes, the therapy helped. But outside of it, if I wasn’t doing the things I was doing, then I wouldn’t have gotten better’ (P26).
	Socialisation	‘And it was early November that this friend wanted to catch up and I was just absolutely terrified. But then slowly as time went on, I would catch up with so-and-so. And then by the end of that year I was involved in a couple of theatre groups and I was actually doing some group activity again’ (P4).
	Education	‘[having independently enrolled themselves in TAFE and work experience] … My TAFE course, I’m so happy that I’m doing it now. I wish I could have done it sooner … I finally found something that works for me’ (P15).
	Employment	‘And then [EIPS] sort of gave me the confidence and the ability to bounce back and say, you know what? I’m going to go again for this job’ (P25).

P1, participant no. 1, etc.; EIPS, Early Intervention Psychosis Services; TAFE, Technical and Further Education.

‘Like, there’s like one I remember seeing one in headspace and it’s like for “out of the box teenagers” or something. And then it’s got like a picture of a skateboard on the cover … And it’s just like I don’t know. It feels like it doesn’t feel like it’s from a relatable standpoint … And that feels kind of patronising, like treating us like teenagers instead of just like people’ (P2).

Where participants did not find formal peer support helpful, some noted the connection formed with informal peers they met in the EIPS groups or hospital, alongside pre-existing relationships with friends or family. A later-stage participant from Sydney (P19), for example, noted:

Participant: ‘Yeah, I didn’t find them [formal peer support] very helpful.’

Interviewer: ‘Yeah, what do you think wasn’t very helpful about it?’

Participant: ‘I think for me, it was just the fact that I knew they were paid to sit and talk to me. So like our case managers and everybody else.’

Interviewer: ‘And so the other girl that you met through this programme who had bipolar, was that just through a group session?’

Participant: ‘I think it was rock climbing, then we started doing cooking classes together, but then we both stopped going. So we just catch up in our own time.’

Interviewer: ‘Yeah. And what aspects of talking to her do you find useful?’

Participant: ‘She’s a very practical person. She very hands on. She’s had the same mental illness. We can joke about the stupid stuff we both did.’

In the later stages of building self-management skills, participants described a shift from needing practical assistance to engaging socially with others or finding a vocation or training to an appreciation that they had the requisite tools to engage in these activities independently. These participants had generally actively engaged with peer support programmes at their EIPS and gone on to create opportunities of their own in education, in work and in life. They generally described an incremental approach where one independent step gave them the confidence to take another:

‘Well, it’s just a gradual process. It started when I did that Technical and Further Education (TAFE) vocational course), then I did a 4-week prac[tical placement], which was helpful in making me feel normal, like just working with people. Then I was applying to jobs for a while and then I got a casual position and then after-school care and then worked out what I was doing. I also went on ketogenic diet, which I’ve been on since mid-December and I’ve lost nearly 30 kilos from that. And then about a month and a half ago, I started learning Japanese, which I’ve been doing now. One thing led to another which gives you the motivation to take on another thing to occupy yourself with’ (P5).

### A framework for understanding the interplay of the four themes in the personal recovery journey

**Fig. 1:**
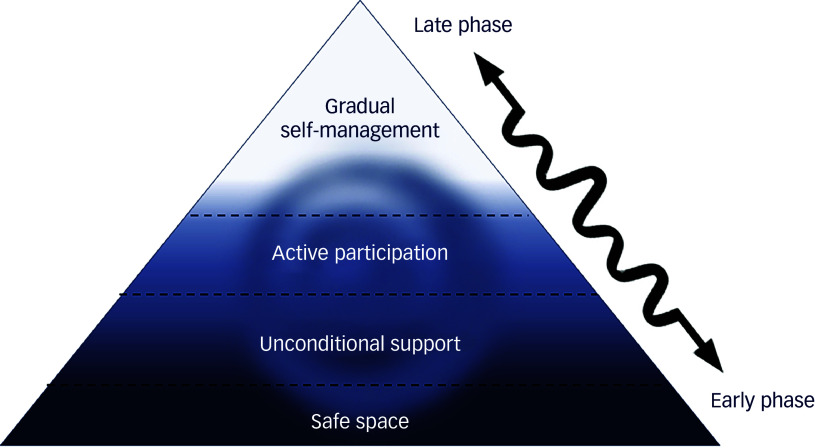
Three foundational themes create the conditions for gradual self-management.

We propose a framework (Fig. 1) for understanding how the four themes we identified affect a given EIPS client’s personal recovery journey: the creation of a safe space, unconditional support and facilitating the active participation of the client in their care were, for our participants, mutually reinforcing factors that are foundational for that client’s gradual self-management. Once participants in our study felt they had a place to go, felt the benefit of a supportive, non-judgemental and encouraging environment and felt like an active participant in their own care, they began to build capacity to manage their own condition and engage, to varying degrees, in social interactions, education and employment. Interviewees who reported experiencing these three things were more likely to go on to describe movement from earlier- towards later-stage self-management.

### Characterising the themes and interplay between them through one participant’s experience

A safe space feels warm and familiar, and has an absence of judgement or a sense of being a burden that means participants can be themselves. It is also, of course, a physical location to which participants can go; probably no small consideration in a young population whose choice of environments can be limited. According to P9:

‘Everyone’s, well, welcoming … happy to see you. Happy to help you as well. Yeah, it was good.’

‘You know, I’m not usually around people that talk about this kind of stuff and I don’t see it on a daily basis, but it was good just to talk about it and let it all out, really.’

‘It was a bit frightening to talk about my situation being a man and being an Indigenous person. So, we don’t really talk about that kind of stuff. It was frightening at first, but then I got used to it just talking about my emotions and mental health.’

Unconditional support is dynamic – it builds on the warmth that participants feel toward their service as a safe space they can go in times of stress or distress, and follows them into the community. The sense that participants have that EIPS staff are on their side when they get in touch with them calls back to their experience of their service as a safe space; when participants reach a point where they are taking the lead in checking in with their service; it calls forward to their active participation in their own care and, ultimately, early recovery. P9 again:

‘I was kind of isolating myself, really, when I was in a bad state but I always reached out to them and they knew, you always knew they were there to help me.’

‘I was able to send my [case worker] a text message or call her up and then see her the next day. But they usually check in once a week or twice a week to see how we’re going and that, so it’s good.’

Active participation – being engaged in decisions around their own management – was an important building block for future decision-making. It was predicated on participant trust (founded by the sense that their service offered a safe space and unconditional support) that their service would support them and their family in times of stress:

‘I was working and, I don’t know, I guess a lot of, under pressure and under stress. That kind of thing and bad sleeping routine as well, always arcs up my paranoia and I had to come in and had a chat with the doctors and that [case worker] as well and they said you can either go hospital or go home and I chose to go home because it’s just more comfortable. It felt safe at home instead of being at a hospital. So, I chose to go home, really. But they gave me, um, support lines and that to call up and support, for mum as well too, to have a better understanding’ (P9).

Clinical engagement in the context of active participation also helped build skills that participants would go on to use independently in their own recovery:

‘I guess everyone’s different, you know. But my friends have always been there and my family. So I guess I talk to them more about what’s going on with me. So that’s a change. And I think that’s the biggest change. I let them know what’s going on’ (P9).

Gradual self-management meant building on the steps participants had taken in the management of their own condition to establish their own connections to work, life and play. In earlier-stage self-management this meant more reliance on their service:

‘… They help me with my depression by giving me medication and we did some exercises to help my anxiety and that. And the medication helps with the paranoia as well. And yeah, they’ve just been supportive by doing levelling exercises that help you better yourself, really, and getting into a routine.’

‘They were very helpful during those times. I quit my job last year and that’s really when I kind of had a relapse. So, they were very helpful to be supportive and that, either to look for a new job or to go into study. So, they were understanding of what I wanted to do’ (P9).

In later-stage self-management, this meant that they took increasing ownership of their recovery:

‘I felt it was probably the first step you’re going to have to do, coz only you can get yourself better, you know. I had to work on my mental health. And it was good coming in here doing the exercises. Yeah, I had to do it myself, really’ (P9).

A hallmark of later recovery was the reorientation of EIPS supports from the basics of recovery to the facilitation of a journey that their clients themselves were driving:

‘They helped me look for jobs, but I wanted to study … and I’m doing these little tasks for helping my anxiety and that. I don’t know, it’s just supportive, really.’

‘Yeah so, my art, started off as just an activity that I do, basically a hobby. And they helped me, well the [employment service] helped me get into uni and [case worker] has been very supportive of helping me in that too, as well.’

‘So, I’m studying at the uni. My first week was last week. So, it was good. It was exciting’ (P9).

## Discussion

This is the first known Australian study to explore in depth the interconnecting factors that affect the personal recovery journey for EIPS clients. These findings reflect the multi-layered nature of recovery, from individual (e.g. self-efficacy) to relational (e.g. therapeutic alliance, peer connection) and structural (e.g. culturally responsive care, service accessibility) levels. Our findings illustrate the importance of foundational conditions in shaping young people’s engagement and recovery experiences, with their recovery not only influenced by their individual experiences but also shaped by their relationships with others and the way services are structured and delivered: ‘Effective self-management is built by small incremental steps: one thing led to another’ (P5).

Much of the support our participants received from their EIPS can be described as self-management interventions, which are designed to educate and equip individuals with the skills to manage symptoms, relapses and overall psychosocial functioning. One well-conducted systematic review and meta-analysis demonstrated that such interventions have a moderate effect on quality of life and functioning, and a significant effect on subjective measures of personal recovery such as self-rated recovery and self-efficacy, compared with treatment as usual.^23^ Our data suggest that the mechanism underlying these changes may be gradual improvement driven by small, incremental successes that build toward skills, knowledge and further improvement – while acknowledging that this is not a linear process. We posit that when young people accessing EIPS report feeling agency in their personal recovery journey, they are more likely to develop – and reinforce – the belief in their own self-efficacy that will enable them to continue to improve and utilise their self-management skills.^24^ Future qualitative studies exploring the interplay between self-efficacy and self-management would be a welcome addition to our understanding of these themes, and may provide policymakers with greater understanding of best-value propositions so that services may orient themselves to provide such interventions more readily.^25^

### Peer support as an enabler of self-management

We found that positive engagement with peer support programmes was often described by young people who had developed their self-management skills considerably. Conversely, participants who reported reluctance to engage with these programmes were also less likely to describe the kind of social interactions, education or employment that would place them towards a later stage of developing self-management skills. Reasons for a reluctance to engage ranged from a general preference to avoiding social interaction to a sense that peer-support programmes were forced and inauthentic. This is a novel finding, and suggests the need for further investigation into the efficacy of peer support programmes in the context of EIPS. We caveat that the association between later-stage self-management and peer-support engagement may be explained by confounders, such as clients with higher baseline function being more likely to engage in peer support activities in the first place. However, a 2018 randomised controlled trial in the UK found that a self-management intervention delivered by peer-support workers significantly reduced rates of readmission for people with severe mental illness to acute care;^26^ our study suggests that novel peer-support interventions such as these, or improvements to the design and implementation of current programmes, may be worthy of study.

### The importance of relatable support at the right time: creating conditions for personal recovery

The capacity and willingness of clients to engage with EIPS services is often affected by the relapsing, remitting course of illness and recovery,^27^ and can include periods of disengagement from services.^28^ Of particular note were the stories of participants who made sudden changes, such as taking on significant work or study commitments, and who simultaneously reduced their contact with their EIPS, culminating in challenges coping with stressors or a subsequent period of relapse. These findings may reflect a need to prepare both services and clients for increased engagement at times of change, even where the change may appear positive. Such preparation is a sensible strategy that builds on best-practice guidelines that recommend intensive mobile outreach when warranted.^1^

For participants from culturally and linguistically diverse backgrounds in particular, programmes were described as more impactful when support was provided by people of the same background who spoke the same language. Wherever possible, creating a diverse EIPS workforce that reflects the population it serves is likely to foster better engagement and align more closely with the goals of client and family support.^1^

Other research has suggested that the lower uptake of recommended interventions in EIPS by young people from minority ethnic backgrounds may ‘reflect clinicians’ ability to offer and explain treatments in a way that appears acceptable and relevant to people from a range of backgrounds, as may the quality and cultural appropriateness of informational materials about treatments, including whether they have been adapted and co-produced with people from the relevant background’.^29^ For culturally and linguistically diverse young people in our study, simple measures, such as information sheets in multiple languages, made participants and their families feel included and provided valuable information that they could approach in their own time. Active involvement of family in decisions around care, especially among culturally and linguistically diverse clients, helped support the young person’s engagement with the service and their recovery. Furthermore, participants who described their families as feeling alienated from the healthcare system often found connection via family peer support. These participants reported that their recovery benefited as a result. This knowledge adds an important nuance to our understanding of how young people with culturally or linguistically diverse backgrounds experience their engagement with EIPS, and indicates opportunities for service improvement for these clients in particular.^30^

### Strengths and limitations of this study

In the context of the extant qualitative literature that has interviewed fewer than 300 young people accessing EIPS, a strength of this study is the relatively large sample size across multiple service locations, with purposive sampling that maximised variation of participant backgrounds.

One limitation introduced by our purposive sampling approach is that clinician nomination of participants (in the context of a service evaluation) may have introduced gatekeeper bias toward the inclusion of EIPS success stories. Another is that participant recollection may have been biased toward especially positive or negative experiences; their experiences are also probably a product of their Australian, chiefly urban, context, which may limit their generalisability. We also acknowledge that participants in this study may reflect a group who are more engaged in, or further along the pathway to, recovery. Previous research (e.g. Chua et al^15^) indicates that disengagement, comorbidities or socioeconomic barriers may shape very different trajectories. Future work should prioritise inclusion of these experiences – particularly through targeted recruitment strategies or linkages to disengagement data.

Our findings regarding support for cultural and linguistically diverse populations are arguably limited by the small number of such participants (*n* = 9). However, this is generally seen as sufficient for substantial coverage of themes^31^ and comprised a third of our sample. Future, more focused, studies on this topic are warranted.

Further to this, in addition to lived experience researchers, we had cultural and linguistically diverse representation in our qualitative team throughout the project who supported the analysis and interpretation of findings. However, although there were three First Nations young people who participated in these interviews, a major limitation of this study is that we did not have a First Nations researcher supporting evaluation design, implementation or analysis.^32^ Future studies would benefit from being resourced to explore the intersection of first-episode psychosis and being a young person who identifies as Aboriginal and/or Torres Strait Islander, through a service provision and service improvement lens.^33,34^

With 20 of the 27 participants’ being more likely to be described as being in later-stage self-management, this may mean that our study preferences the experience of those who are further along in their recovery journey. Future studies may benefit from specific outreach to those who are in the earlier stage, or who have disengaged from their EIPS entirely.

Overall, attention to the foundational conditions that we have outlined (a safe space, unconditional support and active participation in one’s own care) fostered an environment where gradual self-management was possible and a sense of personal recovery more likely. Participants’ movement between earlier- and later-stage self-management did not appear connected to how long they had been EIPS clients, but to their engagement with EIPS in general, and also (for some) to their engagement in formal peer support programmes or utilisation of informal peer support. These journeys through EIPS may also be augmented when clinicians are able to communicate information and provide support in a way that appears acceptable and relevant to people from a range of backgrounds, including those who are culturally or linguistically diverse.

## Supporting information

Caldwell et al. supplementary material 1 Caldwell et al. supplementary material

Caldwell et al. supplementary material 2 Caldwell et al. supplementary material

## Data Availability

Due to the conditions outlined in the study’s consent process, full transcripts cannot be shared, to protect participant anonymity. However, amalgamated data-sets generated and/or analysed during the current study are available from the corresponding author on reasonable request.

## Supplementary material

The supplementary material is available online at https://doi.org/10.1192/bjo.2025.10848
