# Supplementary material for: The clinical and psychosocial journey of young people engaging with early intervention psychosis services: qualitative study
Source: BJPsych Open. 2025 Oct 23;11(6):e252. doi: 10.1192/bjo.2025.10848 (PMC12569612; doi:10.1192/bjo.2025.10848)
Supplement: Caldwell et al. supplementary material 1 — Caldwell et al. supplementary material [file S205647242510848Xsup001.docx]

**Supplementary File 1.** COREQ checklist

| **Number** | **Item** | **Description** | **Page Number** |
| --- | --- | --- | --- |
| 1. | Interviewer | Authors 3 and 9 conducted the interviews with young people accessing EIP services (state and federal). | Methods - data collection |
| 2. | Researcher credentials | Author 1: BA, LLB, MD, MPH, MClinEpi  Author 2: MA, MBBS, MSc, MRCPsych, FRANZCP, PhD  Author 3: BSc, MBBS, M Psychiatry, FRANZCP  Author 4: BScN, MN, PhD  Author 5: BN, MScN, PhD  Author 6: BSc, BEd  Author 7: BSc(Hons), GradCert(Oxon), DCounsPsych  Author 8: BSc, PGDip Psych, MAppSc Health Psych, PhD | Author information, Supplementary File |
| 3. | Occupation | Author 1 is a medical practitioner with experience in qualitative and quantitative research.  Author 2 is an academic psychiatrist.  Author 3 is a consultant psychiatrist who has experience working with EIPS services.  Author 4 is a nurse and qualitative researcher specializing in critical social theory.  Author 5 is a mental health nurse and specialist in qualitative research and qualitative research methodology, with a focus on critical health research, ethnographic theories and methods.  Author 6 is a teacher and lived experience researcher.  Author 7 is a counselling psychologist and Senior Research Fellow in Early Psychosis.  Author 8 is a clinical psychologist and mental health researcher specialising in mental health and culturally and linguistically diverse populations.  Author 9 is a psychologist and researcher specializing in mixed methods research and has Australian and international experience working with EIPS. | Author information, Supplementary File |
| 4. | Gender | Authors 1, 2 and 5 are male.  Authors 3, 4, 6, 7, 8 and 9 are female. | Supplementary File |
| 5. | Experience and training | Authors are experienced and active researchers with expertise in qualitative, quantitative, and mixed methods approaches. Authors have researched and/or published in the broad topic area previously. | Supplementary File |
| 6. | Relationship established | A two-stage consent process was applied, where clinicians briefly described the study to potential participants meeting eligibility criteria gaining consent to refer to the researcher. The clinician scheduled the interview for those expressing interest in participating. Prior to interview, participants had the opportunity to review the participant information and consent forms, and discuss any questions, before giving informed consent. Participants aged 12-18 years required parental/guardian co-consent, with 16-18 year olds parental/guardian consent being subject to clinician advice and state specific laws. | Methods – recruitment and consent |
| 7. | Participants’ knowledge of the interviewer | All interviewers had no previous professional or personal relationship with any of the participants. Co-authors only reviewed de-identified data thus had no personal relationship with, or knowledge of participants. Participants were informed about who the involved researchers were in the Participant Information Statement (PIS). | Methods section, Supplementary File |
| 8. | Interviewer characteristics | The interviewers were conducted by a psychologist and researcher with experience in qualitative and mixed-methods research in Australian and international mental health services including EIPS (Author 9) and a consultant psychiatrist with experience in community-based EIPS, pediatric and adult mental health services and hospital services (Author 3). Interviews were supported by the research team which included Author 6 who is a researcher with lived experience of using EIPS as a client. | Methods section, Supplementary File |
| 9. | Methodological orientation and theory | Reflexive thematic analysis with constructionist epistemology, experiential orientation, predominately inductive approach, and mix of semantic and latent coding was applied. | Methods – analysis |
| 10. | Sampling | To enable participation in the research, clinicians were asked to nominate and invite all clients on their caseload who met eligibility criteria and had capacity to consent. Purposive sampling was used to recruit a diverse sample of the EIPS client population. Representation from special interest groups was prioritised. Special interest group of clients represented various clinical stages (e.g. at-risk, First Episode Psychosis), ages, genders, culturally and linguistically diverse and Aboriginal or Torres Strait Islander backgrounds. Sampling did not require researcher access to client records. The researchers fed back to the coordinating clinicians at each EIPs if there were any recruitment gaps and clinicians checked their entire caseload for eligibility so as to minimise the potential for gatekeeping and bias. | Methods – recruitment and consent |
| 11. | Method of approach | Eligible participants were recruited through clinician referral. EIPS clinicians and managers spoke of the study to potential participants and sent communications to clients advising of the study. Clinicians invited all eligible young people on their caseload who met criteria and had capacity to consent. | Methods – recruitment and consent |
| 12. | Sample size | Semi-structured interviews were conducted with 27 young people accessing EIPS. | Methods – participants |
| 13. | Non-participation | One young person chose not partake in the interview after reading the participant information statement. All interviews that commenced were completed. The number of participants who declined at clinician invitation was not recorded. | Methods – data collection |
| 14. | Setting of data collection | Interviews were conducted face-to-face on the EIPS premises or via telephone between Dec 2019 and May 2020. | Methods – data collection |
| 15. | Presence of non-participants | Two YP requested a support person to be present who also participated in the interviews. | Methods – data collection |
| 16. | Description of sample | A diverse sample of young people and receiving support from Australian state- or federally-funded EIPS.  Participants were young people accessing a participating EIPS. The eligibility criteria included: (1) aged 12-25 years; (2) clinician nominated; (3) minimum two week service engagement; (4) provided parent or guardian consent if aged between 12-15 years, and when advised by the clinician, aged between 16-18 years. | Methods, Table 1. |
| 17. | Interview guide | Interviews were semi-structured. Questions covered: Client experience of coming into the program; Client experience of the program; Client views on the impact of the program on their functional outcomes; Client hospitalisation experience whilst involved in the program; Treatment (medication, CBT, family care); Ongoing community care, mobile outreach and group programs; Family programs and family peer support; Youth participation and peer support program. In order to enhance question relevance, a lived experience researcher who had used EIPS contributed to the interview design. | Methods, Supplementary File 2. |
| 18. | Repeat interviews | No repeat interviews were conducted. | NA |
| 19. | Audio/visual recording | Interviews were audio-recorded. | Methods (data collection) |
| 20. | Field notes | No. | NA |
| 21. | Duration | The average interview duration was 57 minutes. The median interview duration was 55 minutes. | Methods (data collection) |
| 22. | Data saturation | As this study was part of a national evaluation, we did not assess data saturation. While saturation is commonly used in qualitative health research, its applicability to reflexive thematic analysis has been critiqued. In this study, pragmatic sampling targets were guided by evaluation scope and practical constraints, rather than by formal saturation criteria. | Methods (data collection) |
| 23. | Transcripts returned | Transcripts were not returned to participants. | Methods (data collection) |
| 24. | Number of data coders | Data were coded by author 1 and supported by author 9. | Methods (analysis) |
| 25. | Description of the coding tree | Codes, themes, and subthemes were developed by author 1 and sense-checked in regular meetings with supervising author 9. Descriptions of the themes, subthemes and codes were developed and captured in a coding framework facilitated in Nvivo 12 software. | Methods (analysis), Results |
| 26. | Derivation of themes | Themes and subthemes were derived from the data using iterative inductive processes. The theoretical underpinnings of this approach were a constructionist epistemology (what gave meaning and meaningfulness to the participants’ experiences?); an experiential orientation (how did the participant experience a given phenomenon?); The overarching analysis was inductive (a ‘bottom-up’ identification of themes and patterns in the data) using a mix of semantic and latent coding. This analysis was compared alongside previous analysis conducted by our team for the EPYS report and published paper (Milton et al. 2023). These analyses supported reflexivity, comparisons and sharing of diverse perspectives of the research group offered via their various backgrounds, culture, lived experience and professional roles. | Methods, and described in more detail in this supplementary file. |
| 27. | Software | NVivo 12 | Methods (analysis) |
| 28. | Participant checking | Participant checking did not take place. Outside of the lay summary of the findings being returned, there was no formal opportunity for participants to feedback on the findings and recommendations other than contacting the researchers directly. | Not described outside this supplementary file; to be described in the text of our submission to BJ Psych. |
| 29. | Quotations presented | Illustrative quotes from participants were used. Quotations are identified with participant number to ensure anonymity. | Results, Table 2, Table 3. |
| 30. | Data and findings consistent | Data and findings are presented throughout the manuscript. Differences in participants’ experiences were highlighted in results when identified by researchers engaged in analysis. | Results |
| 31. | Clarity of major themes | Reflexive thematic analysis yielded 4 clear major themes that united the underlying concepts in participants’ experiences. | Results, Figure 1, Table 2, Table 3 |
| 32. | Clarity of minor themes | Subthemes were presented throughout. Dictates of space in this paper did not permit discussion of minor themes beyond subthemes in the context of the 4 major themes we identified. | Results, Figure 1, Table 2, Table 3 |
