# Supplementary material for: The clinical and psychosocial journey of young people engaging with early intervention psychosis services: qualitative study
Source: BJPsych Open. 2025 Oct 23;11(6):e252. doi: 10.1192/bjo.2025.10848 (PMC12569612; doi:10.1192/bjo.2025.10848)
Supplement: Caldwell et al. supplementary material 2 — Caldwell et al. supplementary material [file S205647242510848Xsup002.docx]

**Supplementary File 2.** Interview Guide

| **Concept** | **Topic** | **Possible Questions** |
| --- | --- | --- |
| Pathway to care  (access and expectations) | 1. Client experience of coming into the program | 1. *Can you please tell me about what led up to you becoming involved with this program?*  - *Researcher to confirm whether referral to service was via hospital. If yes, what was that experience like for you? If not, can you tell me about the process of coming into the program? Prompt: How long did it take between having some problems and then engaging with hYEPP (headspace Youth Early Psychosis Program)/EIPS?*  1. *What were you hoping to get out of the program? Prompt: What did you think you needed from the program?* |
| Engagement and perception of programs appropriateness | 1. Client experience of the program | 1. *Can you please tell me about the types of problems or issues that the program has helped you to manage?*  - *Prompt: What types of support or care have you received from the program? Are you able to access care when you need it?*  1. *What aspects of the program work well or not so well for you? Prompt: Are you able to access care/support when you need it?* |
| Functional Outcomes associated with the program | 1. Client views on the impact of the program on their functional outcomes | 1. *We are interested in hearing your thoughts on the impact of the hYEPP/EIPS program on different areas of your life. Can you think of anything that has changed for you since accessing the program?*  - *Prompt if requiring further examples: This might include areas of your life such as employment or education, your relationships or social life, your leisure activities, your daily living skills (like looking after yourself, managing your finances, looking after your home)*  1. *For each outcome mentioned ask: Can you tell me what this change means for you personally?*  - *Prompt: is it important or not? Why is that?*  1. *Given these changes, can you tell me how you continue to manage your mental health or any symptoms? Probe: self-management, personal understanding of situation/ diagnosis, support networks.* 2. *How was the hYEPP/EIPS staff/service involved in these changes in your life?* 3. *Were any other services or people involved in these changes?* |
| Hospitalisation experiences | 1. Client hospitalisation experience whilst involved in the program | 1. *Did you have any hospital admissions while receiving hYEPP/EIPS care?*  - *Prompt: If yes explore… how many times were you in hospital? Was it a voluntary or an involuntary admission? What was the impact of this experience on your engagement with services?*  1. *What was the process of going into hospital, being in hospital and transitioning out of hospital like for you?* 2. *What was the support from the hYEPP/EIPS staff like for you during this time?*  - *Prompt: Can you tell me how you found the communication between you, the hospital staff and the hYEPP/EIPS service?* - *Prompt: Can you tell me how you felt about your involvement in your own care?* - *Prompt: What aspects of your care did you focus on with the hYEPP/EIPS service at this time?*  1. *Overall, do you feel that hYEPP/EIPS involvement was helpful or not helpful to you during this time? Why is that?*  - *Prompt: Is there anything the hYEPP/EIPS team did particularly poorly or particularly well?* - *Prompt: Can you think of anything else you would have liked from the hYEPP/EIPS team at this time?*  1. *Have you had any hospitalisation experiences when you were not with the hYEPP/EIPS program? If yes, how do these experiences compare?* 2. *Could you tell us whether you feel that being a part of the hYEPP/EIPS program impacted the length of your hospital stay or your admission to hospital in anyway?* |
| *Specific Fidelity Topics*  *Note: these topics may already have been addressed by participants in response to Question B. If not, these topics may be discussed.* | | |
| Fidelity | 1. Treatment (medication, CBT, family care) | 1. *Can you please tell me about any treatments you were offered or received? (What, When, How, Who) Prompt: How are decisions about your treatment made?* 2. *What aspects of treatment worked well or not so well for you?* |
|  | 1. Ongoing community care, mobile outreach and group programs | 1. *Can you tell me about the kinds of service and care you have received? (Who, What, When, Why) Prompts: Have you ever needed crisis support or care after hours? Have you attended any group programs?* 2. *What aspects of these types of services work well or not so well for you?* |
|  | 1. Family programs and family peer support | 1. *Have any of your family, carers or friends participated in any of the family programs or family peer support programs? (What, When, How, Who)* 2. *Can you tell me how their involvement in the program has been helpful to you, or not?* |
|  | 1. Youth participation and peer support program | 1. *Can you please tell me about any involvement you had with the youth participation and peer support programs? (What, When, How, Who)* 2. *Do they sound interesting or appealing to you?* 3. *What aspects of these programs work well or not so well for you?* |
